# High prevalence and expression of antiseptic resistance genes among infectious t037/ST239 methicillin-resistant *Staphylococcus aureus* (MRSA) strains in North Khorasan Province, Iran

**DOI:** 10.22038/IJBMS.2022.63780.14055

**Published:** 2022-06

**Authors:** Hamed Ghasemzadeh-Moghaddam, Amir Azimian, Ghasem Bayani, Vahid Dashti, Sara Nojoomi, Nojoomi Shirazi, Akbar Solati, Alex Van Belkum

**Affiliations:** 1 School of Medicine, North Khorasan University of Medical Sciences, Bojnurd, Iran; 2 Vector-borne Diseases Research Center, North Khorasan University of Medical Sciences, Bojnurd, Iran; 3 Imam Hassan Hospital, North Khorasan University of Medical Sciences, Bojnurd, Iran; 4 Open Innovation & Partnerships, BaseClear, Sylviusweg 74, 2333 BE Leiden, The Netherlands

**Keywords:** Benzalkonium chloride, Chlorhexidine gluconate, Disinfection, Iran, Methicillin-resistant - Staphylococcus aureus, Nosocomial Infection

## Abstract

**Objective(s):**

*Staphylococcus aureus* is an important infectious agent and the majority of methicillin-resistant *S. aureus *(MRSA) infections are of nosocomial origin. To define the level and distribution of antiseptic resistance among infectious* S. aureus *strains we studied MRSA and methicillin-susceptible *S. aureus *(MSSA) isolates collected from different infection sites in an assortment of patients.

**Materials and Methods:**

*S. aureus* isolates were investigated for* in vitro* susceptibility to antiseptic agents and detection of *qacA/B*, *smr*, *vanA*, and *mecA* genes.

**Results:**

Among the *S. aureus* isolates we studied, 25 and 41 were MRSA and MSSA, respectively. The mean of minimum inhibitory concentrations (MICs) for benzethonium chloride (BTC) among MRSA was statistically significantly higher than for MSSA (26 µg/ml versus 11.7 µg/ml, *P*=0.003) while there was no significant difference between MRSA and MSSA for benzalkonium chloride (BKC) and chlorhexidine digluconate (CHG). The *qacA/B* genes were carried in 68% of the MRSA and 58.2% of MSSA (*P*=0.601), while *smr* was carried in 39% of MRSA and 29.3% of MSSA strains (*P*=1.000). In 15 out of 25 cases, MRSA ST239 with spa types t037, t030, and t7688 was isolated from the infection site with 86.6% of them carrying a resistance gene (*qacA/B* or *qacA/B* + *smr*).

**Conclusion:**

The frequent presence of antiseptic resistance genes and a consequently elevated MIC against antiseptics among ST239 MRSA emphasizes the importance of mandatorily monitoring MRSA for effective infection control.

## Introduction

*Staphylococcus aureus* is an important infectious agent, and the majority of methicillin-resistant *S. aureus* (MRSA) related infections are of nosocomial origin. MRSA is well known to be resistant to beta-lactam antibiotics and other antibiotics as well (1). Also, vancomycin resistance among *S. aureus* is becoming an increasingly important issue (2) reported from different parts of the world (3, 4), including Iran (5). Despite the selective use of antibiotics and additional prevention strategies, MRSA-related nosocomial infections are still a major problem. Disinfection and decolonization may fail in the hospital setting because of possible resistance to antibiotics and antiseptic agents that may lead to increased severity of staphylococcal infections and extension of associated clinical and epidemiological problems. Infection control management strategies including decontamination of colonized body sites using antiseptic agents such as chlorhexidine and quaternary ammonium compounds have been shown to reduce the risk of invasive nosocomial infections (6, 7). 

Damaging the phospholipid bilayer and cytoplasmic membrane disruption are anti-bacterial characteristics of chlorhexidine and quaternary ammonium compounds. Resistance to these compounds is primarily mediated by multidrug efflux pumps encoded by two families of PCR non-differentiable genes (*qacA*, *qacB* and *qacC*, *qacD*, and *ebr*) known as the *qacA/B* and *smr* families, respectively (8). These plasmid-located, transferable gene families confer high and low-level resistance to antiseptics, respectively (9, 10).

In the current study, we define the level and distribution of antiseptic resistance among infectious *S. aureus* strains. We studied the MRSA and MSSA isolates collected from different infection sites of hospitalized patients in Imam Hassan Hospital of Bojnurd, the main referral hospital center in the North Khorasan Province in Iran. 

## Materials and Methods

***Study samples***

The current study used the *S. aureus* isolates collected from infected hospitalized patients in Imam Hassan Hospital, the main tertiary care referral hospital in North Khorasan Province, Iran. The study duration was from January to December 2020. 

All isolates were identified to the species level in the hospital laboratory and were reconfirmed in the microbiology laboratory at the Faculty of Medicine by standard Gram staining, catalase testing, mannitol testing, and tube coagulation assays. All isolates were further confirmed as *S. aureus *by *Sa442* PCR (11). The *S. aureus* isolates were confirmed as MRSA by oxacillin and cefoxitin susceptibility testing according to Clinical and Laboratory Standard Institute (CLSI) guidelines (12) and *mecA* PCR (13) (sequence of primers and used programs deposited in the supplementary information file).

All isolates were then stored at -30 °C in Trypticase Soy Broth (TSB), supplemented with 20% glycerol (HiMedia, India). Chromosomal DNA was extracted using a commercial DNA extraction kit (Poyagene Azma, Iran) according to the manufacturer’s instructions. Purified DNA was used for molecular investigations.

***Antiseptic susceptibility testing***

Susceptibility to quaternary ammonium compounds such as benzethonium chloride (BTC), benzalkonium chloride (BKC), and biguanide compounds such as chlorhexidine digluconate (CHG; Sigma-Aldrich, Steinheim, Germany) was determined using the Mueller–Hinton broth microdilution method (BMD) (8). A concentration of 1.25 to 1250 µg/ml of antiseptic was used for measuring the MICs.

***Detection of genes encoding antibiotic resistance, antiseptic resistance, and regulators***

All isolates were screened for the presence of *qacA/B*, *smr* (*qacC*, *qacD*, and *ebr*), and *vanA* genes as described before (5, 8). Multiplex PCR for detection and characterization of the staphylococcal cassette chromosome mec (*SCCmec*) and accessory gene regulator (*agr*) typing were performed by protocols described before (13-15) (sequence of primers and used programs deposited in the supplementary information file). 

***Spa*** **typing**

All isolates were subjected to *spa* typing using previously described methods (16) and exploiting the services provided by Ridom SpaServer (https://spaserver.ridom.de/). Related multi-locus sequencing typing (MLST) results were extracted from Ridom SpaServer if available. 

***Statistics***

Differences between MSSA and MRSA strains were tested for significance using Fischer exact test and independent samples t-test. *P*-values<0.05 were considered significant.

## Results

Resulting from the 76 reported *S. aureus* infections in the study period, 66 *S. aureus* isolates from different infection sites were available. These included 25 MRSA and 41 MSSA strains. *S. aureus* isolates were obtained from different clinical samples such as tracheal aspirates (n = 27), blood samples (n = 14), wound swabs (n = 12), urines (n = 6), and others (n = 7). The mean age of the infected patients was 48 (4–85) years comprising 38 (57.6%) males and 28 (42.4%) females (Table 1). The vast majority of strains originated from the Intensive Care Unit (ICU) (n=25, 37.8%) and the emergency ward (n=14, 21.2%). The highest rate of MRSA infection (n=13, 52%) was documented among ICU patients. All emergency ward infections agents were MSSA (n=14, 34.1%) (Table 1). Tracheal aspirate yielded the highest number of *S. aureus* (n=27, 40.9%), followed by blood (n=14, 21.1%), and wound swabs (n=12, 18.1%) while most lung infections were apparently caused by MRSA (n=15, 60%) (Table 1).

There was a significant difference in the MICs for BTC between MRSA strains (26 µg/ml) and MSSA strains (11.7 µg/ml) (*P=*0.003) (Table 2). No significant differences in MICs for BKC (*P=*0.200, 35.5 µg/ml versus 20.6 µg/ml) and CHG (*P=0.460*, 272 µg/ml versus 222 µg/ml) among MRSA and MSSA were observed (Table 2 and the supplementary data file).

Among the *S. aureus *isolates, 69.7% harbored at least one resistance gene while no significant difference between MRSA and MSSA was observed (72% MRSA versus 68.3% MSSA, *P=*0.7899) (Table 3). The incidence of the *qacA/B *genes among MRSA (68%) was higher than MSSA (58.2%) but again there was no statistically significant difference (*P=*0.6016). No difference in the distribution of the *smr* gene between MRSA and MSSA was observed either (*P=*1.000). The *qacA/B* genes presented among n=41/66 isolates (62.1%) in comparison with the *smr* gene that was detected among n=30/66 of *S. aureus *isolates (30.3%) (Table 3). No *vanA* gene responsible for vancomycin resistance and phenotypic resistance to vancomycin was detected.

The most common *agr* type among MSSA (n=33/41, 80.5%) and MRSA (23/25, 92%) was *agr I. *Among *agr I* type isolates, the *qacA/B* gene was detected in 34.7% (8/23) of MRSA and 63.6% (21/33) of MSSA isolates with no significant difference (*P=*0.2449). Among the MRSA, *SCCmec* *III* (11/25, 44%) and *SCCmec IV* (7/25, 28%) were the most common types (Table 3).

Among the MRSA strains, the mean MICs against three antiseptic reagents were higher in t037, t030, and t7688 (n=15) ST239 strains (Table 4). The majority of t037/ST239 (n=11/12) and the only t7688/ST239 strains were isolated from tracheal aspirate cultures among ICU ward patients. Wound cultures (n=4/8) and urine cultures (n=2/8) were the common clinical sources for t230/ST45 MRSA strains from the patients in different wards (Table 4 and the supplementary information file).

Among MRSA strains, the majority of (t037, t030, and t7688)/ST239 strains (86.6%) were *qacA/B* gene-positive, and five t037/ST239 strains were positive for *qac* and *smr* simultaneously (Table 4). Only one t037/ST239 strain harbored no antiseptic resistance gene at all. The prevalence of resistance genes among other MRSA strains was 40% and 30% for *qacA/B* and *smr*, respectively (Table 4).

A review of the *SSCmec* types encountered in our study is presented in Table 4. The majority of MRSA strains were typed as *SCCmecIII* (n=11) when 9 of them were t037/ST239 strains (Table 4). The only t7688/ST239 MRSA strain was *SCCmecXI*, and it harbored the *qacA/B* gene as well (Table 4). 

*Spa* typing of MSSA strains revealed more genetic diversity in comparison with *Spa* typing of MRSA. The distribution of resistance genes among MSSA strains was more diverse than among MRSA (supplementary information file). 

**Table 1:** Patients' demographic data and *Staphylococcus aureu****s*** infection sites

*S. aureus*	Gender				Age		Ward									Site of infection				
	Male	Female		Mean range		ICU		INT	HEART	EMRG	NERO	SW	INF	PEDI	LC		BC	WC	UC	Other			
	No. (%)			Years		No. (%)									No. (%)								
MSSA	19 (46.3)		22 (53.7)	49 (4-85)		12 (29.3)		4 (9.7)	1 (2.4)	14 (34.1)	3 (7.3)	4 (9.7)	2 (4.8)	1 (2.4)	12 (29.3)		12 (29.3)	6 (14.6)	4 (9.7)	7 (17.1)		
MRSA	19 (76)		6 (24)	54 (9-83)		13 (52)		4 (16)	0	0	2 (8)	1 (4)	4 (16)	1 (4)	15 (60)		2 (8)	6 (24)	2 (8)	0	
Total	38 (57.6)		28 (42.4)	48 (4-85)		25 (37.8)		8 (12.1)	1 (1.5)	14 (21.2)	5 (7.5)	5 (7.5)	6 (9.1)	2 (3)	27 (40.9)		14 (21.1)	12 (18.1)	6 (9.1)	7 (10.6)		

ICU: intensive care unit, INT: internal medicine, Heart: cardiology, EMRG: emergency, SW: special ward, INF: infectious diseases, PEDI: pediatric, LC: tracheal aspirate culture, BC: blood culture, UC: urine culture, WC: wound culture

**Table 2:** MIC of antiseptic compounds among MSSA and MRSA strains

MIC	BTC		BKC		CHG	
*S. aureus*	Mean (range) µg/ml	*P*	Mean (range) µg/ml	*P*	Mean (range) µg/ml	*P*
MSSA	11.7 (2.5–39)	0.003	20.6 (2.5–39)	0.200	222 ( 39–625)	0.460
MRSA	26 (5–78)		35.5 (5–155)		274 (78–625)	
Total	26.5 (2.5–625)		35.5 (2.5–625)		255 (39–625)	

MSSA: methicillin-sensitive *Staphylococcus aureu****s, ***MRSA: methicillin-resistant *Staphylococcus aureus, *BTC: benzethonium chloride, BKC: benzalkonium chloride, CHG: chlorhexidine digluconate, MIC: minimum inhibitory concentration, *P*: independent samples t-test

**Table 3:** Distribution of resistance and regulatory genes among MSSA and MRSA strains

*S. aureus*	Genes													
	Resistance				*agr*					*SCCmec*				
	*qacA/B*	*smr*	Sum*		I	II	III	IV	NT	I	II	III	IV	XI
	No. (%)				No. (%)					No. (%)				
MSSA	24 (58.5)	12 (29.3)		28 (68.3)	33 (80.5)	1 (2.4)	4 (9.8)	0	3 (7.3)	-	-	-	-	-
MRSA	17 (68)	8 (32)		18 (72)	23 (92)	0	2 (8)	0	0	4 (16)	2 (8)	11 (44)	7 (28)	1 (4)
Total	41 (62.1)	20 (30.3)		46 (69.7)	56 (84.8)	1 (1.5)	6 (9.1)	0	3 (4.5)	4 (16)	2 (8)	11 (44)	7 (28)	1 (4)

MSSA: methicillin-sensitive *Staphylococcus aureu****s, ***MRSA: methicillin-resistant Staphylococcus aureus,*: Some strains had two genes simultaneously, NT: Not typeable, *smr*: identical to *qacC*, *qacD*, and *ebr*, *qacA/B*: identical to *qac A* and *qacB*

**Table 4:** Typing results, resistance genes distribution, and MIC against antiseptics among MSSA and MRSA strains

MRSA (25)	*Spa *type(No.)/CC	MLST		Resistance genes (No.)										BTC		BKC		CHG			
		ST/CC		*SCCmec*A		*qac*		Total		*smr*		Total		mean MIC µg/ml (range)					
	t037(12) /37, t30(2)/37	239 /8	III(10), I(3), II(2)		12				5				27.7(9.7–78)		43.7(9.7–155)		305(78–625)	
	t7688 (1)/24	239 /8	*SCCmec* C		1		17		0		8		39		39		155	
	t230(8)/S	45/45	IV(7), I		2		68%		2		32%		21.3(5–78)		20.7(5–78)		214(78–625)	
	t1741(2)/S		III		2				1				332(39–625)		332(39–625)		468(310–625)	
MSSA(41)	t790(9),t1149(7),t701(4), t571(3)				21				12				11.7(2.5–39)		20.6(2.5–39)		222(39–625)	
	t1077(3),t1077(3), t5598(2)						24				12							
	t2622(2),t127, t706, NT (2)						58.5%				29.3%							
	t30(3), t12(2) ,t05	239,30,22			3				0									
	

S: Singleton , CC: Clonal cluster, ICU: Intensive care unit, Nero; Nephrology, MIC: minimum inhibitory concentration

MSSA: methicillin-sensitive *Staphylococcus aureu****s, ***MRSA: methicillin-resistant *Staphylococcus aureus, *BTC: benzethonium chloride, BKC: benzalkonium chloride, CHG: chlorhexidine digluconate, LC: tracheal aspirate culture, BC: blood culture, UC: urine culture, WC: wound culture

## Discussion

*S. aureus*, especially MRSA, is a well-known source of nosocomial infection in Iran (17). Staphylococcal infections are occurring from both endogenous and exogenous sources. Even though *S. aureus* preferentially colonizes the anterior nares, which may lead to endogenous infection, such colonized patients remain at risk of exogenous infections as well (18). Antiseptic resistance among MRSA has been reported in Iran before (19), but we here present the first study that compared the actual MIC values in combination with documented presence of antiseptic resistance genes in strains isolated from infections in Iran.

The highest measured MICs for antiseptic agents (625 µg/ml) that we documented among *S. aureus* strains included in the present study are still lower than the recommended concentration of use (2000, 1000, and 5000 µg/ml for BKC, BTC, and CHG, respectively) (19). t037, t030, and t7688 (ST239/CC8) strains were reported as successful MRSA and MSSA strains in the hospital setting of Iran (17, 20-26) and Asia (27). The elevated prevalence of antiseptic resistance genes and the concurrent increase in MIC against antiseptic agents among mentioned infectious strains poses a clinical dilemma. The possible use of a subinhibitory dose of disinfectants in the hospital setting may increase the chance of further enhancing those MICs. This could happen through dilution of cleansing liquids or other mechanisms such as up-regulation of efflux pumps (28). In consequence, it may also lead to a higher chance of exposing patients to these successful and potentially invasive MRSA isolates in hospitals. 

In the current study, *qacA/B *genes, which provide resistance to a broader range of biocides than *smr* (29-31), were the predominant resistance genes as was reported before in the United States (32), European countries (33), Japan (34), Iran (19), China (35), and Hong Kong (36). The incidence of *qacA/B* reported here is higher than reported results from 11 Asian countries (38.5%) (37) including Korea (59%) (38). It is lower than what was reported before in Malaysia (83.3% and 69.4%) (39, 40). The detected prevalence of the *qacA/B* gene is almost the same as in 12 European countries (62.6%) (33). 

This is the first report of t7688/ST239 strains from infections in our study region. t7688/ST239 was reported earlier as a livestock-related clone (25) and it was registered previously as a colonizing strain from Iran in Ridom spa server (https://spaserver.ridom.de/). t7688/ST239 was isolated before from the hands of staff nurses and the hospital environment (41), and it also revealed itself as a successful cause of infection which was isolated from different infection sites in hospitals in Iran (24-26). It seems that t7688/ST239 is one of the successful clones among ST239/CC8 MRSA strains in Iran. The presence of antiseptic resistance genes followed by elevated MICs against antiseptic agents verifies the importance of detailed monitoring of all MRSA strains, colonizers, or infections from different clones.

*SCCmecIV* was reported among t7688/ST239 MRSA strains in some Iranian studies before (25, 41), whereas the current study revealed for the first time the presence of *SCCmecXI* in this clone. We have seen no prior report of *SCCmecXI* among ST239/CC8 strains. The *SCCmecXI* was reported among CC130 strains from human and livestock sources from European countries including the United Kingdom and Denmark (42) (43). The majority of other reports involved different wild animals such as European otters and a European brown hare from Austria (44) as well as European hedgehogs from Sweden (45). Since then, zoonotic transmission (46) and its association with invasive disease (47) have been reported. There is no report for detecting the *SCCmecXI *gene among strains harboring the human-specific immune evasion cluster (IEC) (48). The presence of this *SCCmec* type among ST239/CC8 strains that reported positive for IEC genes in Iran (49, 50) and other countries (51, 52) warns of the possibility of the presence of wild animal-related *S. aureus* clones in regional hospitals and transferring other virulence genes from them to human-adapted strains. 

## Conclusion

The results of the current study highlight the value of close monitoring of MRSA strains in the hospital setting for the presence of antiseptic resistance genes and their associated phenotypic resistance. Typing and screening the clones circulating in a hospital is strongly recommended for better understanding the epidemiology of antiseptic resistant MRSA strains, especially *S. aureus *clones that are also common among wild animals.

## Authors’ Contributions

HGM Performed conceptualization, project administration, and wrote the original draft; AA Helped with investigation and resources; VD, SN, and SS Provided investigation; AS Helped review and edit; AVB Helped write, review, and edit, and gave scientific advice.

## Funding Information

This work received no specific grant from any funding agency.

## Ethical Approval

Sample collection was performed according to the rules and regulations set by the Ethical Committee of North Khorasan University of Medical Sciences (project number: IR.NKUMS.REC.1394.027).

## Conflicts of Interest

The authors declare that there are no conflicts of interest.
